# Exogenous indole promotes florfenicol tolerance in *Edwardsiella tarda*

**DOI:** 10.1080/21505594.2026.2620188

**Published:** 2026-01-21

**Authors:** Yu Zheng, Luhua Fu, Zhuoying Cao, Ting Zhang, Jiao Fei, Ming Jiang, Yuying Zhou, Zhi Shi, Yubin Su

**Affiliations:** a Department of Immunology and Microbiology & Institute of Medical Microbiology, MOE Key Laboratory of Viral Pathogenesis & Infection Prevention and Control (Jinan University), National Engineering Research Center of Genetic Medicine, Guangdong Provincial Key Laboratory of Bioengineering Medicine, College of Life Science and Technology, Jinan University, Guangzhou, China; b Department of General Practice, The First Affiliated Hospital of Xiamen University, School of Medicine, Xiamen University, Xiamen, China; c State Key Laboratory of Tropical Oceanography, South China Sea Institute of Oceanology, Chinese Academy of Sciences, Guangzhou, China; d College of Life Sciences, Hunan Normal University, Changsha, China; e Department of Cell Biology and Institute of Biomedicine, National Engineering Research Center of Genetic Medicine, MOE Key Laboratory of Tumor Molecular Biology, Guangdong Provincial Key Laboratory of Bioengineering Medicine, College of Life Science and Technology, Jinan University, Guangzhou, China

**Keywords:** *Edwardsiella tarda*, antibiotic tolerance, indole, metabolic reprogramming, reactive oxygen species

## Abstract

Bacterial metabolism is important for antibiotic resistance and tolerance. However, the impact of indole on bacterial metabolism and antibiotic efficacy has not been fully elucidated. In this study, we investigated the effect and specific mechanism of exogenous indole on the antibiotic susceptibility of *Edwardsiella tarda*, a common pathogen in freshwater and marine fish farming. We found that exogenous indole promoted *E. tarda* tolerance to the antibiotic florfenicol, and reprogrammed the *E. tarda* metabolome. A total of 108 metabolites were detected, including 66 differential metabolites that regulate various metabolic pathways, such as the tricarboxylic acid (TCA) cycle and nucleotide metabolism. Exogenous indole disrupted the TCA cycle in *E. tarda* by increasing the intracellular NADH contents and activating the respiratory chain to increase the reactive oxygen species levels, thereby increasing the intracellular Fe^2+^ content to activate the Fenton reaction, which in turn promotes the oxidative stress response. Furthermore, indole inhibited antibiotic entry into the cell and activated efflux pumps to reduce the intracellular antibiotic content, ultimately promoting antibiotic tolerance. In vivo, exogenous indole compromised the ability of florfenicol to protect fish survival and eliminate pathogenic bacteria. These results shed light on the metabolic changes induced by indole and suggest future directions for addressing antibiotic tolerance and clinical infections of *E*. *tarda* in aquaculture. This study serves as a reminder of the adverse effects of combining antibiotics with metabolites in aquaculture.

## Introduction

The gram-negative bacterium *Edwardsiella tarda* (genus: *Edwardsiella*) exhibits characteristics that are typical of *Enterobacteriaceae* [1]. Owing to its abundance in aquatic environments, *E. tarda* is a common pathogen in freshwater and marine fish farming, targeting a wide range of fish species and leading to huge economic losses in industry [2]. Although it is rarely pathogenic to humans, *E. tarda* can result in severe, disseminated infections in individuals with predisposing conditions [3].

Antibiotics are used to prevent and control bacterial infections; however, their overuse induces bacterial tolerance, which often precedes resistance development during bacterial evolution [4–6]. Unlike bacterial resistance, bacterial tolerance enables populations to survive antibiotics without altering the minimum inhibitory concentration and is not caused by genetic mutations [7]. Bacterial tolerance supports the continued survival of bacterial populations, thereby increasing the likelihood of rare resistance mutations arising. Additionally, by increasing the number of surviving bacteria, tolerance reduces the chance of resistance mutations being lost during antibiotic exposure. Overall, this poses a substantial challenge to health practitioners, human health, and aquaculture, and highlights the need for a deeper understanding of the antibiotic tolerance mechanisms of pathogens is important [8].

The discovery that bacterial environments and pathways confound antibiotic efficacy has changed our understanding of the modes of action of bactericidal antibiotics. Antibiotics trigger the upregulation of bacterial stress responses, such as the stringent response, in bacteria [9]. In addition, an antibiotic treatment affects bacterial redox homeostasis and energy-generating pathways such as the tricarboxylic acid (TCA) cycle [10,11]. Evidence has shown that the metabolic environment of microorganisms can confound their susceptibility to antibiotics [9,11–13]. Cellular respiration is a fundamental metabolic process for living organisms. Lobritz et al. showed that increasing the basal respiration rate of *Escherichia coli* at the genetic level significantly increased the efficacy of bactericidal antibiotics [14]. Furthermore, several recent studies have demonstrated that regulating metabolic pathways in bacteria can alter their susceptibility to antibiotics. For example, enhancing purine metabolism restores bacterial susceptibility to antibiotics [15]; exogenous uracil addition enhanced cellular respiration and reprogrammed the metabolome of methicillin-resistant *Staphylococcus aureus* to increase bacterial susceptibility to antibiotics [16]; and arginine restriction induced antibiotic tolerance in *S. aureus* by inhibiting protein synthesis [17]. These results indicate that exogenous supplementation with specific metabolites can regulate bacterial sensitivity to antibiotics in different metabolic states. Thus, the rational use of metabolic regulation has potential for overcoming antibiotic tolerance.

Indole, recognized as a typical N-heterocyclic aromatic pollutant in industrial and agricultural wastewaters, also serves as a bioactive signaling molecule with extensive environmental presence. This endogenous bacterial metabolite, produced by over 85 bacterial species, significantly influences bacterial physiology and metabolism [18,19]. Indole affects antibiotic resistance and tolerance in both indole-producing and non-indole-producing bacteria [20,21]. For example, indole reduced the virulence of *S. aureus* [18] and promoted biofilm formation in *Pseudomonas aeruginosa* [22]. Indole signaling in *E. coli* inhibited efflux pumps and subsequently modulated tolerance in other bacteria [23]. Indole signaling also inhibited phage attack to protect *P. aeruginosa* [24]. Other research has shown that indole affects the bacterial response to antibiotics, reactive oxygen species (ROS), and other stressors, increasing antibiotic resistance and tolerance by activating efflux pumps and inducing oxidative stress [25–27]. Furthermore, indole induced antibiotic tolerance in *P. fluorescens* via its inductive transcriptional regulator EmhR [28]. Despite these findings, the metabolic changes induced by the exogenous addition of indole and its mechanism of action on antibiotic efficacy are not fully understood.

*Edwardsiella* species include *E. ictaluri*, *E. piscicida*, *E. tarda*, *E. anguillarum*, and *E. hoshinae*. Among these, *E. piscicida* is a well-recognized and important fish pathogen. In the present study, we focus on *E. tarda*, which is one of the serious threats affecting worldwide aquaculture. We hypothesize that exogenous indole promotes antibiotic (florfenicol) tolerance in *E. tarda* through metabolic reprogramming. To test this hypothesis, we use metabolomic analysis to explore how exogenous indole affects the metabolic state of *E. tarda* ATCC15947 and contributes to antibiotic tolerance in *E. tarda*.

## Results

### Exogenous indole promotes florfenicol tolerance in *E. tarda*

**Figure 1.**
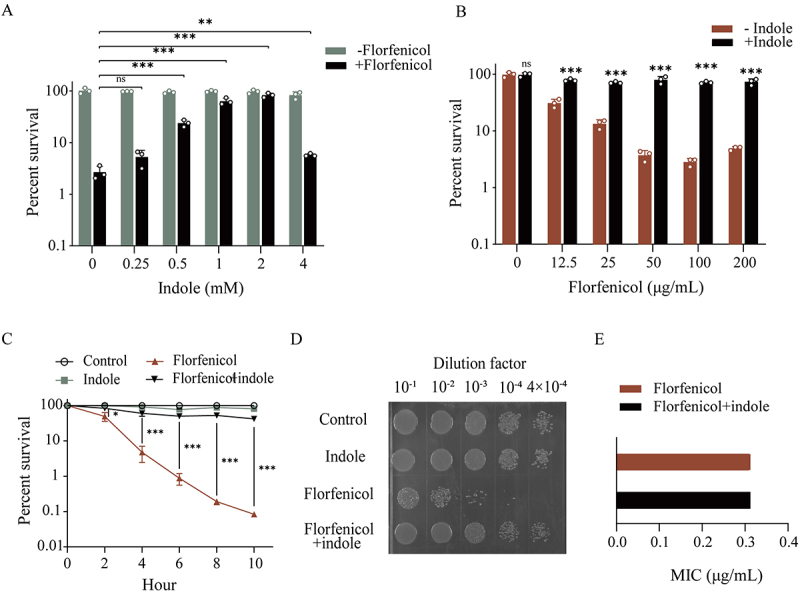
Indole promotes ATCC15947 tolerance to florfenicol. (A) Combined bactericidal results of different concentrations of indole and 100ug/mL of florfenicol. (B) Combined bactericidal results of different concentrations of florfenicol and 2 mM indole. (C) Temporal gradient bactericidal results of 100 μg/mL florfenicol and 2 mM indole. (D) Dot-plate plots of 100 μg/mL florfenicol and 2 mM indole. E. Minimum inhibitory concentration of florfenicol of *E. tarda* after indole or/and florfenicol treatment change in inhibitory concentration. Data are presented as mean ± SEM (*n* = 3 biological replicates). ns stands for no significant difference.

After exogenous indole treatment, *E. tarda* ATCC15947 exhibited reduced sensitivity to the antibiotic florfenicol, as indicated by an increase in the bacterial survival rate. This effect was concentration-dependent, and at a concentration of 2 mM indole, it was observed to maximally reduce antibiotic sensitivity (Figure 1(A)). Previous studies have reported that indole compounds accumulate to millimolar concentrations in the gastrointestinal tract [29], highlighting indole as a relatively abundant small molecule metabolite in the intestine. Treatment with a combination of 2 mM indole and 100 μg/mL florfenicol achieved the best improvement in survival rate (Figure 1(B)) and showed time-dependent effects (Figure 1(C)). The specific dot-plate effect is shown in Figure 1(D). In addition, compared to florfenicol alone, exogenous indole enhanced bacterial motility and reduced the inhibition zone size of *E. tarda* ATCC15947 when added together with florfenicol, as evidenced by the bacteria’s spread (Figure S1A and B). To determine whether indole treatment promoted resistance or tolerance to florfenicol in *E. tarda*, we determined the minimum inhibitory concentration (MIC). The MIC values were consistent with and without indole treatment, suggesting that increased bacterial survival following indole treatment was due to enhanced bacterial tolerance rather than drug resistance (Figure 1(E)). These results demonstrated that indole can remarkably promote florfenicol tolerance in *E. tarda*.

*p < 0.05. **p < 0.01. ***p < 0.001.

In addition to florfenicol, indole also promoted *E. tarda* ATCC15947 tolerance to erythromycin, chloramphenicol, doxycycline and ciprofloxacin whose MICs did not change (Figure S1C and D). Moreover, indole treatment had a similar effect on florfenicol tolerance in *Vibrio alginolyticus* ATCC33787, *V. parahaemolyticus* ATCC15947, and *Aeromonas hydrophila* Ah-HN1 (Figure S1E), suggesting that this effect may be prevalent among bacteria in aquaculture.

### Exogenous indole alters E. tarda metabolome

Metabolomics was used to analyze the metabolic changes occurring in *E. tarda* ATCC15947 cells after exogenous indole addition. The untreated group was used as the control, and organisms from the control and indole-treated groups were collected for metabolomic analysis. The global metabolite heatmap is shown in Figure S2A. A total of 108 metabolites were identified and categorized into six groups: amino acids, nucleosides, carbohydrates, fatty acids, lipids, and others, which accounted for 21%, 26%, 12%, 17%, 11%, and 13% of metabolites, respectively (Figure S2B). The proportions of upregulated and downregulated metabolites in each category are shown in Figure S2C. Amino acids, nucleosides, and carbohydrates had 16, 10, and 11 downregulated metabolites, respectively, and the remaining three categories had six downregulated metabolites each. The number of upregulated metabolites in these categories was 7, 18, 3, 12, 6, and 7, respectively.

**Figure 2.**
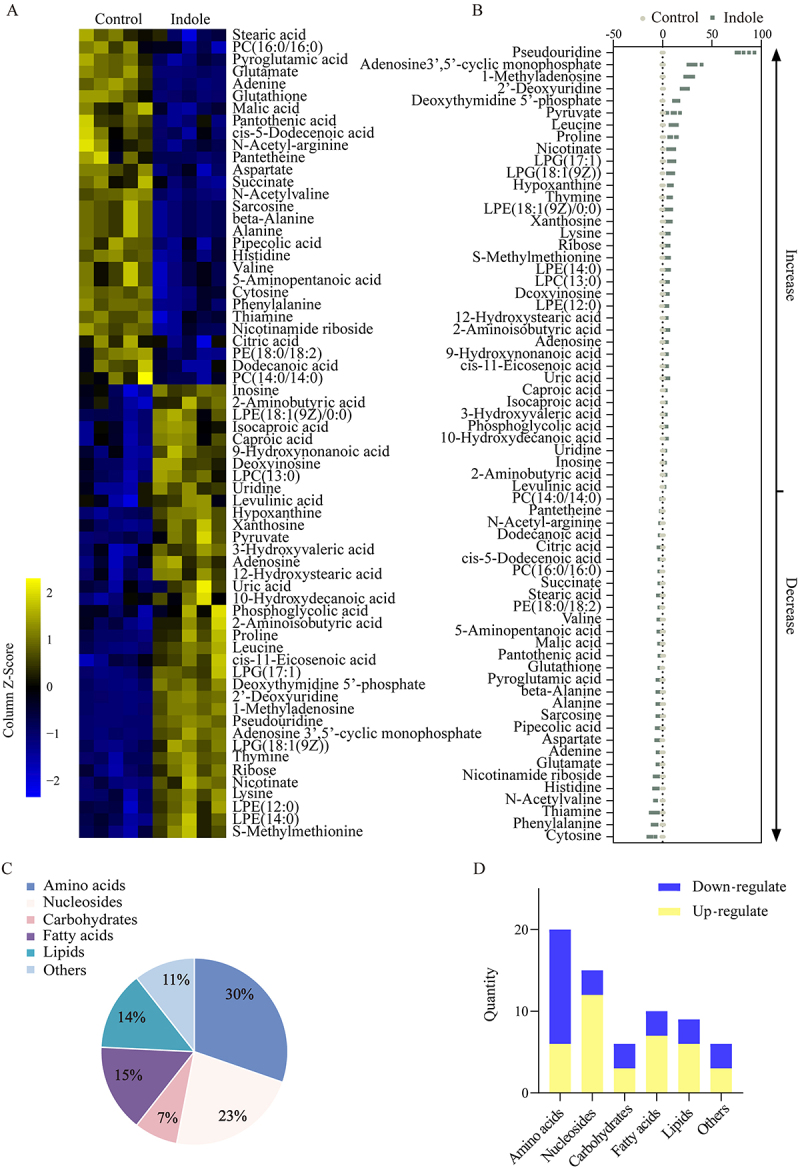
Differential metabolite analysis. (A) Differential metabolite heat map. Yellow represents up-regulated metabolites and blue represents down-regulated metabolites. (B) Differential metabolite z-value map. (C) Differential metabolite category and percentage. (D) Number of up-regulated and down-regulated metabolites in the differential metabolite category.

A total of 66 metabolites showed differential abundance after treatment with indole (Figure 2(A)). Subsequent Z-value analysis of these 66 derived differential metabolites depicted the metabolite changes (Figure 2(B)), revealing with 37 upregulated and 29 downregulated metabolites after indole treatment. Among them, the top five up-regulated compounds were pseudouridine, adenosine-3“,5”-cyclic monophosphate, 1-methyladenosine, 2”-deoxyuridine, and deoxythymidine-5”-phosphate. The top five down-regulated compounds were cytosine, phenylalanine, thiamine, N-acetylvaline, and histidine. These differential metabolites were categorized into six groups: amino acids, nucleosides, carbohydrates, fatty acids, lipids, and others, which accounted for 30%, 23%, 7%, 15%, 14%, and 11% of metabolites, respectively (Figure 2(C)). The number of upregulated and downregulated metabolites in each category is shown in Figure 2(D), which demonstrated that amino acid metabolites were predominantly downregulated, and other metabolites showed equal numbers of upregulated and downregulated metabolites, whereas the remaining categories of metabolites were predominantly upregulated.

### Exogenous indole reprograms *E. tarda* metabolome

**Figure 3.**
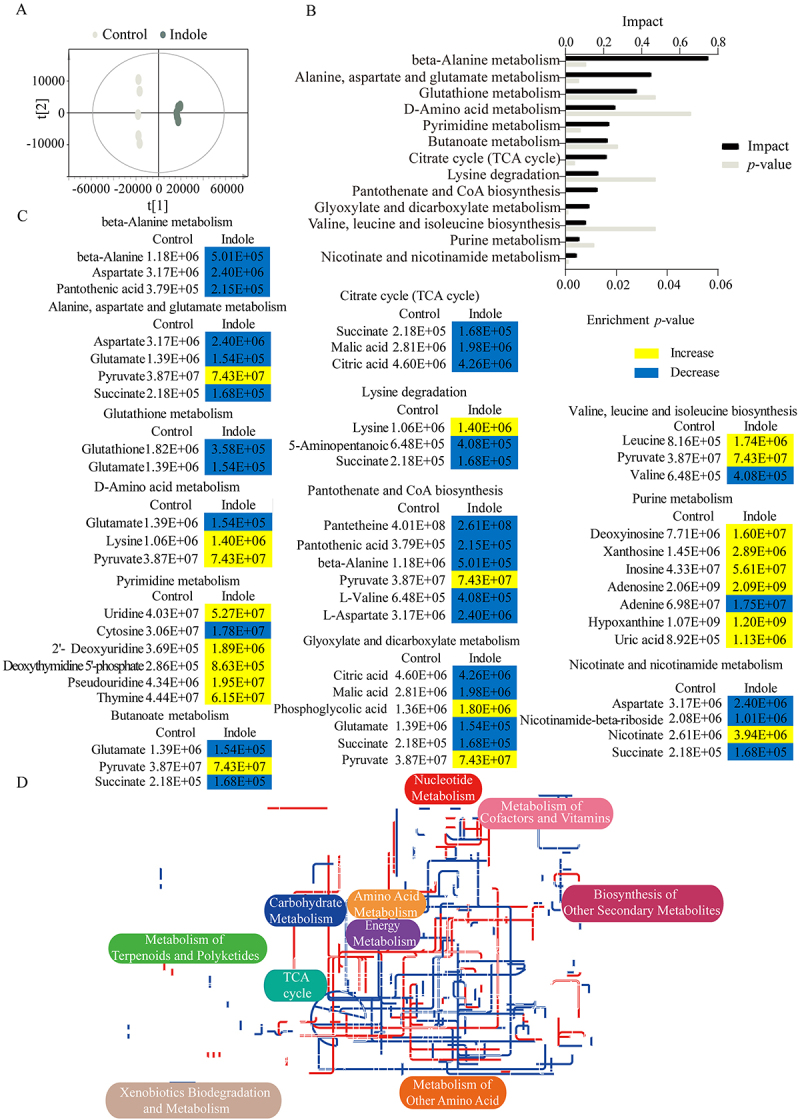
Pathway enrichment and differential substances. (A) Score matrix integration plot. (B) Significant enrichment of metabolic pathways after indole treatment. (C) Changes in metabolites involved in differential metabolic pathways. Yellow and blue colors represent increased and decreased metabolites, respectively. (D) Changes in iPath pathway maps after indole treatment. Red color represents up-regulated pathways and blue color represents down-regulated pathways.

Principal component analysis was performed on the differential metabolites, where the t [1] factor distinguishes between the two groups, and the t [2] factor distinguishes between biological replicates within the groups (Figure 3(A)). S-plot testing of the differential metabolites revealed 31 biomarkers (Figure S3A). Of these, 22 were up-regulated and nine were down-regulated (Figure S3B and C). Pathway enrichment analysis revealed 13 pathways (Figure 3(B)), with the top five ranked as follows, from high to low impact: β-alanine metabolism; alanine, aspartate and glutamate metabolism; glutathione metabolism; D-amino acid metabolism; and pyrimidine metabolism. Among the differential metabolites involved in the significantly enriched pathways, those in the TCA cycle, which is the central carbon metabolism pathway, were predominantly downregulated (Figure 3(C)). Subsequent analysis using ipath 3.0 (Figure 3(D)) revealed down-regulation of the TCA cycle in *E. tarda* ATCC15947 cells after the exogenous indole addition. These results suggest that indole reprogrammes the *E. tarda* metabolome and disrupts the TCA cycle.

### Exogenous indole activates the respiratory chain

**Figure 4.**
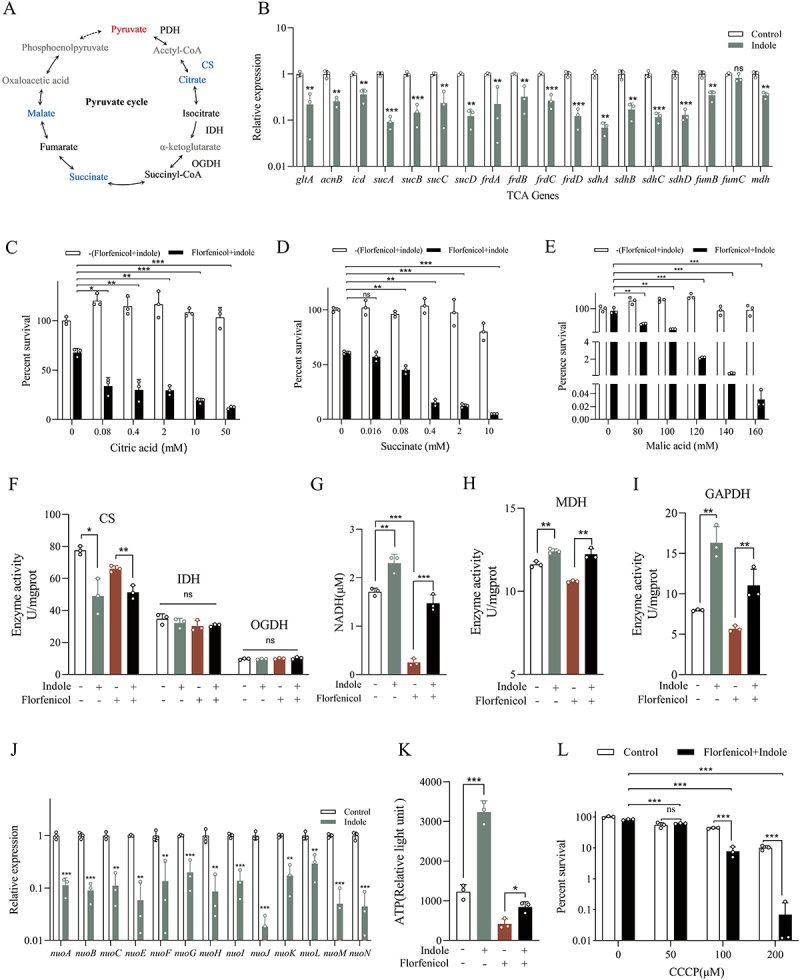
Indole disrupts the TCA cycle and activates the respiratory chain. (A) Diagram of P cycle mechanism. Red represents up-regulated metabolites, blue represents down-regulated metabolites, black represents unchanged, and gray represents undetected metabolites. CS for citrate synthase, OGDH for α-ketoglutarate dehydrogenase, ICDH for isocitrate dehydrogenase. (B) Effect of exogenous indole addition on the gene expression of TCA cycle. (C–E) Effect of exogenous citric acid, succinic acid, and malic acid on florfenicol combined with indole. (F) Effects of indole on the enzyme activities of three key enzymes of the TCA cycle. (G) Changes in NADH dehydrogenase gene expression in the respiratory chain in response to indole treatment. (H) Changes in NADH dehydrogenase gene expression in the respiratory chain in response to indole treatment. (I) Effect of exogenous indole on malate dehydrogenase (MDH) activity in the TCA cycle. (J) Effect of exogenous indole on the activity of glyceraldehyde 3-phosphate dehydrogenase (GAPDH). (K) Changes in intracellular ATP content after indole treatment. (L) Bactericidal effect of the respiratory chain inhibitor CCCP on the combination of 100 μg/mL florfenicol and 2 mM indole.

The TCA cycle is part of the pyruvate cycle (P cycle) [30]. Among P cycle substances, pyruvate was upregulated by indole treatment, whereas citrate, succinate, and malate were downregulated (Figure 4(A)). The three downregulated metabolites were identified as components of the TCA cycle. We analyzed the expression of TCA cycle-related genes and found that indole inhibited their expression, except for *fumC*, which remained unchanged (Figure 4(B)). Subsequently, we added the three metabolites together with indole, all of them significantly restored the sensitivity of *E. tarda* ATCC15947 to florfenicol (Figure 4(C–E)). We measured pyruvate dehydrogenase (PDH) activity, a key enzyme in the P cycle, but found no changed (Figure S4A). Additionally, we measured the activities of the three key TCA cycle enzymes: citrate synthase, isocitrate dehydrogenase, and α-ketoglutarate dehydrogenase, and observed that only citrate synthase activity was inhibited, while the others remained unchanged (Figure 4(F)). Interestingly, we found that, in the metabolomic analysis, other metabolites in the TCA cycle, such as oxaloacetate, isocitrate, α-ketoglutarate, and fumarate, despite their unchanged abundance, also restored sensitivity to florfenicol (Figure S4B), suggesting that metabolite-controlled regulation of bacterial susceptibility to antibiotics is complex.

Data are presented as mean±SEM (*n*=3 biological replicates). ns stands for no significant difference. *p < 0.05. **p < 0.01. ***p < 0.001.

Since these metabolites are involved in NADH production, changes in their abundance may affect intracellular NADH levels; therefore, we next measured the intracellular NADH content. Surprisingly, intracellular NADH content increased significantly after indole treatment, whereas florfenicol treatment decreased this levels. Indole addition reversed the NADH decrease caused by florfenicol (Figure 4(G)). Through measurements of NADH-producing enzymes malate dehydrogenase (MDH) and glyceraldehyde-3-phosphate dehydrogenase (GAPDH), we found that indole significantly increased their enzyme activities (Figure 4(H,I)). Furthermore, the expression levels of NADH dehydrogenase-related genes were determined, showing that indole reduced their expression (Figure 4(J)). NADH is converted into ATP via the electron transport chain [31], and our results demonstrated that indole treatment increased intracellular ATP contents (Figure 4(K)). Subsequently, adding CCCP to inhibit the respiratory chain significantly reduced *E. tarda* ATCC15947’s tolerance to florfenicol (Figure 4(L)). These results suggest that exogenous indole disrupts the TCA cycle but increases intracellular NADH contents, which enhances respiratory chain activity and ATP production.

### Exogenous indole increases ROS levels to induce oxidative stress

**Figure 5.**
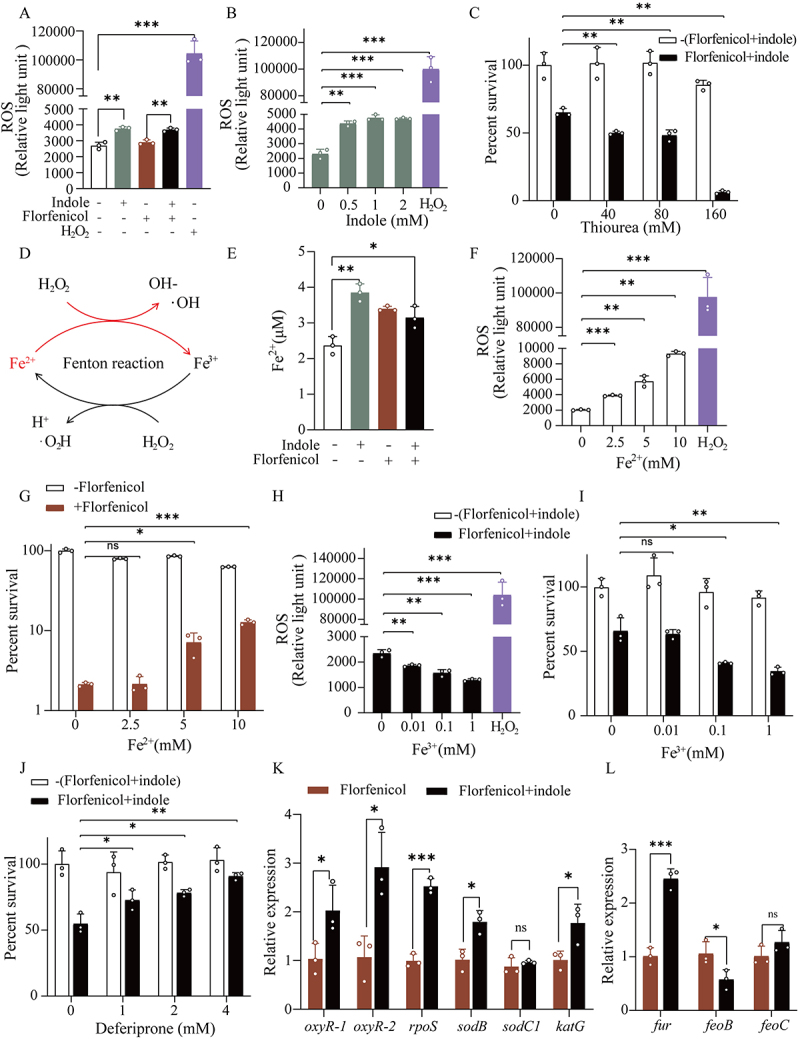
Oxidative stress induced by indole activation of the Fenton reaction. (A) Changes in intracellular ROS content in response to indole treatment. Positive control was 30% hydrogen peroxide (H_2_O_2_). (B) Dependence of intracellular ROS content on indole concentration. (C) Diminution of the effect of the ROS scavenger thiourea on the restoration of the combination of indole and florfenicol. (D) Fenton reaction diagram. (E) Changes in intracellular Fe^2+^ content in response to indole treatment. (F) Dependence of intracellular ROS content on the concentration of FeSO_4_ (Fe^2+^). (G) Different concentrations of FeSO_4_ (Fe^2+^) in response to florfenicol of combined sterilization results. (H) Effect of coincubation of trivalent ammonium ferric citrate (Fe^3+^) with 2 mM indole and 100 μg/mL florfenicol on intracellular ROS content. (I) Influence of trivalent ammonium ferric citrate (Fe^3+^) on the effect of the combined group of indole and florfenicol. (J) Deferiprone enhances the effect of the combined restoration of indole and florfenicol. (K) Elevation of ROS activates expression of oxidative stress-related genes. (L) Intracellular regulation of ferrous ion content-related gene expression changes.

NADH acts as an electron carrier, providing electrons to the respiratory chain for transfer, which then undergoes oxidative phosphorylation to generates superoxides [32]. Therefore, changes in NADH content can also affect ROS levels. In this study, intracellular ROS levels were significant increased after indole treatment (Figure 5(A)) and showed a concentration-dependent relationship with indole content (Figure 5(B)). To verify whether the increased ROS caused bacterial tolerance, we added thiourea, a ROS scavenger, to the incubation medium (Figure 5(C)). *E. tarda* ATCC15947 tolerance to florfenicol significantly decreased after scavenging ROS. As ROS are associated with the Fenton reaction (Figure 5(D)) [33], we measured intracellular Fe^2+^ and found that the addition of exogenous indole caused a significant increase in intracellular Fe^2+^ (Figure 5(E)). Moreover, after adding different Fe^2+^ concentrations instead of indole to *E. tarda* ATCC15947 cells, we observed a concentration-dependent increase in intracellular ROS levels with Fe^2+^ (Figure 5(F)), as well as a gradual increase in the bacterium survival rate (Figure 5(G)). Furthermore, adding Fe^3+^ along with indole to reverse the Fenton reaction led to a significant decrease in both the intracellular ROS levels and bacterial survival rate (Figure 5(H,I)).

Data are presented as mean±SEM (*n*=3 biological replicates). ns stands for no significant difference. *p < 0.05. **p < 0.01. ***p < 0.001.

For further validation, we added desferrioxone to scavenge intracellular Fe^3+^. The results showed that the survival rate of *E. tarda* ATCC15947 cells increased with the decreasing Fe^3+^ contents (Figure 5(J)). Finally, we measured the expression of genes related to ROS-induced oxidative stress and Fe^2+^ regulation. We found that *oxyR-1*, *oxyR-2*, and *rpoS*, key regulators involved in the oxidative stress response, and the corresponding regulated genes, *katG* and *sodB*, were significantly upregulated, whereas *sodC1* expression was unchanged (Figure 5(K)). The increase in intracellular Fe^2+^ content increased expression of the iron-uptake regulator *fur* and decreased expression of the major Fe^2+^ transporter gene *feoB*, whereas the expression of the other Fe^2+^ transporter gene *feoC* was unchanged (Figure 5(L)). These results show that exogenous indole induces an increase in intracellular ROS levels and promotes the Fenton reaction, which in turn stimulates the oxidative stress response, triggering changes in the expression of relevant genes, thereby enabling *E. tarda* survival.

### Exogenous indole promotes efflux pumps to reduce the intracellular antibiotic content

**Figure 6.**
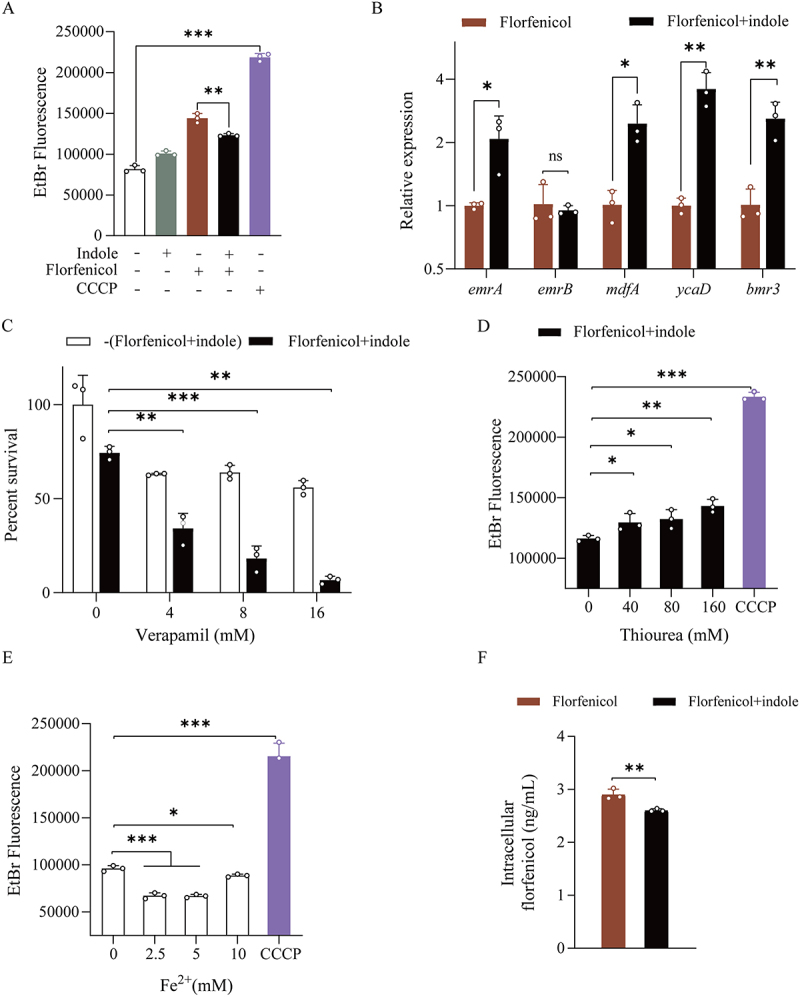
Indole promotes efflux pump expression. (A) Ethidium bromide assay of efflux pump activity. Positive control was 200 μM CCCP. (B) Changes in bacterial efflux pump gene expression in response to indole treatment. (C) Efficacy of efflux pump inhibitor verapamil in attenuating the restorative effect of the combination of 2 mM indole and 100 μg/mL florfenicol. (D) and (E) Effect of thiourea/ferri ion on efflux pump activity. (F) Changes in intracellular antibiotic content after indole treatment.

Bacteria have many efflux pump systems in their cell membranes, which they can activate to pump out antibiotics and increase their survival rate. The fluorescent dye ethidium bromide was used to measure the efflux pumping activity of bacteria, which was inversely correlated with the fluorescence intensity. The efflux pump activity of *E. tarda* treated with indole and florfenicol was greater than that of bacteria treated with florfenicol alone (Figure 6(A)). Indole treatment significantly increased expression of the efflux pump genes *emrA*, *mdfA*, *ycaD*, and *bmr3* by 1.88-, 3.00-, 3.59-, and 3.06-fold, respectively. However, *emrB* expression was unchanged (Figure 6(B)). Next, verapamil, a common efflux pump inhibitor, was incubated with the combined group (indole and florfenicol); the results showed that inhibition of the efflux pump activity significantly attenuated the increased *E. tarda* tolerance induced by indole treatment (Figure 6(C)). As enhanced ROS levels can activate the bacterial efflux pump [34], we incubated the ROS scavenger thiourea with the combined group; the results showed that the efflux pump activity decreased with increasing thiourea concentration (Figure 6(D)). As our previous results indicated that increased ROS following indole treatment also increases intracellular Fe^2+^ contents, we added different Fe^2+^ concentrations to *E. tarda* ATCC15947 and tested its efflux pump activity. As expected, an increase in the Fe^2+^ content activated the bacterial efflux pump (Figure 6(E)). We subsequently measured the intracellular florfenicol content, which was significantly decreased in the group with indole addition (Figure 6(F)). These results demonstrate that indole can activate bacterial efflux pumps by increasing ROS levels, which promotes the efflux of antibiotics, thereby increasing bacterial tolerance.

Data are presented as mean±SEM (*n*=3 biological replicates). ns stands for no significant difference. *p < 0.05. **p < 0.01. ***p < 0.001.

### Exogenous indole alters membrane permeability to inhibit antibiotic influx

**Figure 7.**
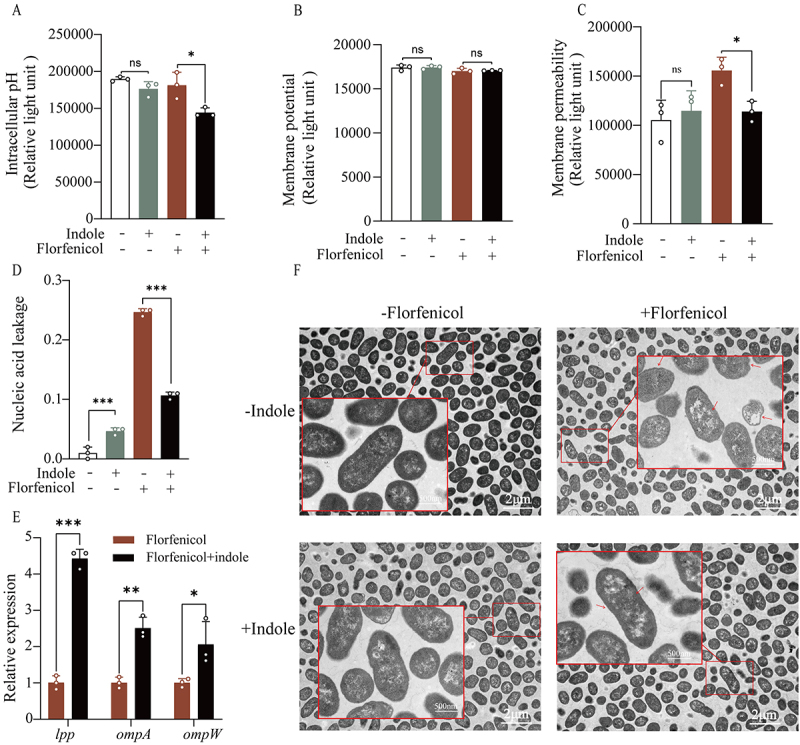
Indole decreases membrane permeability. (A) and (B) Changes in ΔpH inside and outside bacteria in response to indole treatment and in bacterial membrane potential △φ. (C) Effect of exogenous indole on bacterial membrane permeability. (D) changes in leakage of bacterial intracellular material after treatment with exogenous indole. (E) the gene expression of bacterial outer membrane proteins. (F) The effect of indole on the thickness of the cell membrane was observed using transmission electron microscopy. The concentrations of indole and florfenicol were 2 mM and 100 μg/mL, respectively, and the magnifications of the overall and localized images were 4, 000x and 20, 000x, respectively.

The proton motive force (PMF) comprises the transmembrane proton gradient (∆pH) and electric potential (∆φ). The ∆pH value in the combined group was significantly decreased compared to that in the florfenicol-only group (Figure 7(A)), whereas ∆φ was unchanged (Figure 7(B)). Subsequently, we measured the membrane permeability of the four treatment groups: that of the combined group was significantly reduced compared with that of the florfenicol-only group (Figure 7(C)). The results of the cytoplasmic leakage assay, which indirectly represents the integrity of the cellular membrane (Figure 7(D)), agreed with the results of membrane permeability. The expression of two typical outer membrane protein-encoding genes, *ompA* and *ompW*, was significantly increased, whereas the expression of *lpp*, a gene encoding a major bacterial lipoprotein, showed a similar significant increase (Figure 7(E)). These results were further verified by microscopy (Figure 7(F)), which showed smoother bacterial cell membranes in the combined group, with no excessive wrinkles and more electron-dense regions than those in the florfenicol-only group. These results suggest that combined treatment with indole and florfenicol affects the membrane permeability of *E. tarda*.

Data are presented as mean±SEM (*n*=3 biological replicates). ns stands for no significant difference. *p < 0.05. **p < 0.01. ***p < 0.001.

### Exogenous indole attenuates the protective efficacy of florfenicol against fish infections

**Figure 8.**
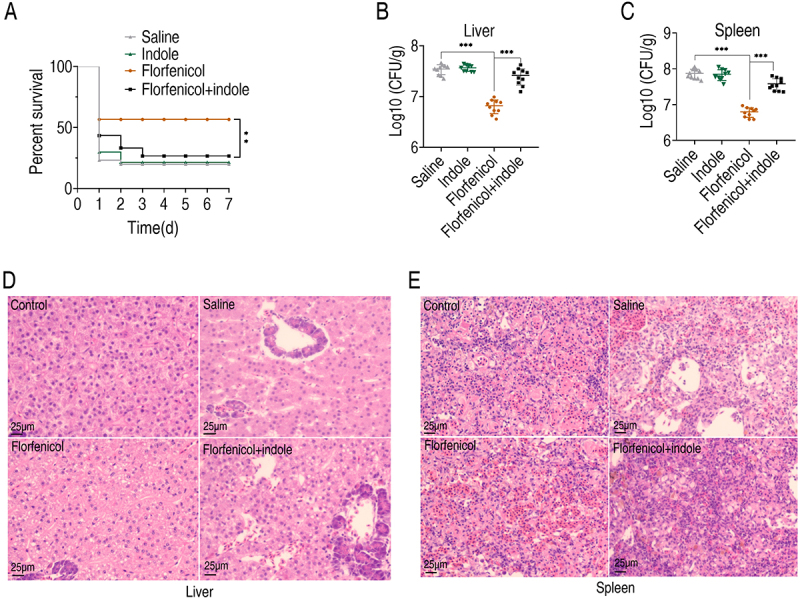
Indole reduces survival of Nile tilapia and clearance of *E. tarda* with florfenicol. (A) Survival rate of Nile tilapia treated with 10 mg/kg indole and/or 15 mg/kg florfenicol. (B) and (C) Changes in the number of bacteria in the liver and spleen of Nile tilapia under different treatments. (D) and (E) Staining effect of liver and spleen sections under different treatments. The control group represented Nile tilapia not infected with *E. tarda*, and the other groups represented Nile tilapia infected with *E. tarda*.

Given that indole enhances the in vitro tolerance of *E. tarda* to florfenicol, we further investigated its impact in vivo. As *E. tarda* is a prevalent pathogen causing infections in various fish species, we established a Nile tilapia infection model. After 24-h bacterial infection, fish were treated with saline, indole, florfenicol, or a combination of florfenicol and indole. The results revealed no significant difference in survival rates between the saline and indole groups, with both groups exhibiting approximately 20% survival. In contrast, florfenicol treatment improved survival to 55%. However, the florfenicol + indole group exhibited a progressive decline in survival, dropping to 25% by day 3 and stabilizing thereafter (Figure 8(A)). In bacterial-susceptible organs, including the liver and kidney, colony-forming unit counts demonstrated that florfenicol treatment reduced bacterial loads by nearly one order of magnitude. In contrast, florfenicol + indole treatment led to a rebound in bacterial numbers (Figure 8(B,C)). Histopathological analysis of tissue sections further confirmed these findings. Although florfenicol treatment restored cellular morphology to levels comparable to those in the uninfected controls, both the saline and florfenicol + indole groups exhibited extensive vacuolation, indicating significant tissue damage (Figure 8(D,E)). Collectively, these in vivo results demonstrate that indole not only fails to improve fish survival or bacterial clearance but also compromises the protective effects of florfenicol.

All data are shown as SEM ± means, **p < 0.01, ***p < 0.001 as determined by one-way ANOVA.

## Discussion

Antibiotics are used to treat bacterial infections; however, the problem of bacterial resistance to antibiotics has exacerbated over the decades as the global use of antibiotics has significantly increased [35]. Drug-resistant bacteria pose a substantial threat to human health, and bacterial tolerance to antibiotics can severely impact the treatment of bacterial infections, potentially leading to treatment failure and recurrence of chronic infections [36]. Bacterial tolerance is considered an adaptive feature that enables bacteria to survive antibiotics without developing resistance, but represents an inevitable stage in the evolution of bacteria toward drug resistance [4]. For example, *P. aeruginosa* tolerance to antibiotics has been shown to promote the development of resistance [37]. Therefore, understanding the mechanisms of phenotypic antibiotic tolerance is essential for reducing bacterial resistance.

Metabolites are products of bacterial metabolic processes that play an important role in bacterial functions; therefore, metabolomic techniques have become an important tool for studying bacterial tolerance. Previous research has shown that changes in amino acid metabolism can increase tolerance to antibiotic treatment in *Mycobacterium abscessus* [38]. The exogenous addition of metabolites changes the metabolic state of bacteria, which in turn affects their sensitivity to antibiotics [39,40]. For example, metabolites such as proline and glutamine have been shown to alter bacterial metabolism and restore bacterial sensitivity to antibiotics [41,42].

In this study, we found that indole significantly increased *E. tarda* ATCC15947 survival in the presence of antibiotics. We further compared its metabolic changes with and without the exogenous addition of indole using liquid chromatography – mass spectroscopy. Indole reprogrammed the bacterial metabolism, with differential metabolites detected accounting for approximately 61.1% of all metabolites; these were predominantly amino acids and nucleosides. In addition, indole modulated several metabolic pathways, such as β-alanine, alanine, aspartate, glutamate, and glutathione metabolisms, and disturbed P and TCA cycles. Our results suggest that the disruption of the TCA cycle plays an important role in the indole-induced tolerance of *E. tarda* to florfenicol. After treatment with indole, MDH activity increased resulting in an increase in the intracellular NADH contents. However, the activities of two of the three key enzymes in the TCA cycle were unchanged, and the citrate synthase enzyme activity decreased, suggesting that indole disrupted the TCA cycle. Previous research has also shown that bacterial tolerance to antibiotics can be induced by inhibiting the TCA cycle [43–45]. This study demonstrated that the TCA cycle was reactivated by exogenous supplementation with metabolites from the TCA cycle, thereby reversing indole-induced antibiotic tolerance in *E. tarda*. These findings have important implications for solving the problem of indole-induced bacterial tolerance.

In addition, because of the increase in intracellular NADH contents resulting from indole-induced reprogramming of the *E. tarda* ATCC15947 metabolome, the ATP content correspondingly increased, contributing to an increase in intracellular ROS levels in the bacterium. Gram-negative bacteria, are more tolerant to oxidative stress [46]. Although this may seem to contradict our hypotheses, as elevated ROS levels lead to bacterial cell death in most cases, non-lethal ROS may lead to the development of bacterial antibiotic resistance [47–49] and tolerance [50]. Increased ROS levels cause destabilization of intracellular iron-sulfur clusters, which in turn generates more •OH via the Fenton reaction [51]. In this study, we found that increased levels of Fe^2+^ in *E. tarda* activated the Fenton reaction and, due to the activation of intracellular iron-uptake regulators, inhibited the ferrous iron transporter gene *feoB* through activation of intracellular iron-uptake regulators, thereby reducing external Fe^2+^ uptake. The increase in intracellular ROS activated oxidative stress-related regulators (*oxyR-1*, *oxyR-2*, and *rpoS*) in *E. tarda* and subsequently activated their respective regulated genes (*katG* and *sodB*). *oxyR* controls the scavenging of intracellular peroxides via *katG* expression [52–54], whereas *rpoS* controls the expression of superoxide dismutations via *sodB*, which encodes superoxide dismutase that scavenges the superoxide anion [55,56]. Some studies have found that ROS can induce bacterial drug resistance by activating drug efflux pumps [57,58]. We discovered that ROS activated the expression of genes related to drug efflux pumps, including *emrA*, *mdfA*, *ycaD*, and *bmr3*, which decreased the intracellular levels of florfenicol and increased the survival rate of *E. tarda*. Although florfenicol alone reduces NADH and ATP levels without affecting ROS levels, the exact mechanism remains unclear. Moreover, indole increases NADH, ATP, and ROS levels. This indicates that indole plays a significant role in the process, though the underlying mechanism warrants further investigation.

The electrochemical proton gradient across the cytoplasmic membrane is the PMF, which includes ∆pH and ∆φ and drives important intracellular processes [59,60]. In this study, we found that indole treatment caused a decrease in ∆pH, whereas ∆φ remained unchanged, leading to a decrease in the PMF, which in turn reduced florfenicol uptake by the bacterial strain, as also demonstrated for the antibiotics aminoglycoside [61] and β-lactam antibiotics [40]. Gram-negative bacteria have a unique outer membrane structure. In addition to dissipating bacterial PMF, indole activated the outer membrane protein genes *ompA* and *ompW* in *E. tarda*, thereby ensuring its survival. Outer membrane proteins can help bacteria tolerate harsh environmental conditions and fight the threats posed by antimicrobial compounds [62]. *ompA* and *ompW* encode outer membrane proteins; OmpA maintains the integrity of the cell membrane and OmpW is involved in the efflux process [58,63]. Furthermore, the expression of *lpp*, a gene encoding the bacterial lipoprotein Lpp, was significantly increased by indole treatment. Lpp is ubiquitous in bacteria, and plays an important role in maintaining integrity of the bacterial outer membrane; the loss of this protein decreases membrane integrity, leading to reduced bacterial stiffness and preventing bacterial sensing and stress responses [64,65].

**Figure 9.**
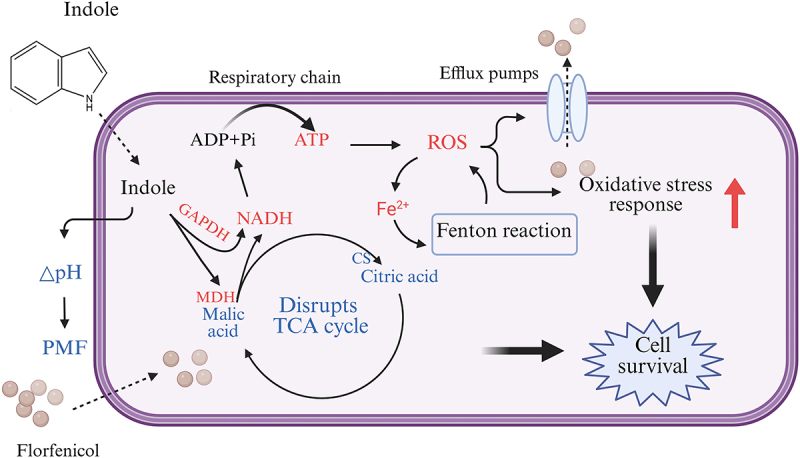
Proposed mechanism of indole-promoted tolerance to florfenicol in *E. tarda*. Exogenous indole reprogrammed the metabolome by increasing intracellular NADH levels through activation of MDH and GAPDH, disrupting TCA cycle, activating the respiratory chain, and increasing ROS levels in vivo in *E. tarda* ATCC15947 to induce oxidative stress and promote efflux. Indole also reduces membrane permeability, decreases intracellular antibiotic levels, and ultimately promotes antibiotic tolerance in *E. tarda* ATCC15947.

In summary, our findings revealed that indole enhances antibiotic tolerance in *E. tarda* under in vitro conditions and attenuates the therapeutic efficacy of antibiotics in fish in vivo. Mechanistically, exogenous indole reprogrammes *E. tarda* ATCC15947 metabolism, increasing intracellular NADH contents by activating MDH and GAPDH while disrupting the TCA cycle. Elevated NADH activates the respiratory chain to increase the ROS levels, which in turn increases antibiotic efflux and decreases membrane permeability to reduce the intracellular antibiotic contents, ultimately promoting antibiotic tolerance in *E. tarda* (Figure 9). This study elucidates the metabolic changes induced by indole and serves as an important reminder that antibiotics used to treat pathogens in aquaculture may have adverse effects if used in combination with some metabolites.

Red for up-regulation, blue for down-regulation.

## Materials and methods

### Bacterial strains and culture conditions

*E. tarda* ATCC15947 strain was obtained from Associate Professor Chao Wang, Shandong Institute of Freshwater Fisheries, Jinan. *V. alginolyticus* ATCC33787 was kindly donated by Prof. Xuanxian Peng from Sun Yat-sen University. *A. hydrophila* strain Ah-HN1 was gifted by Associate Prof. Xianliang Zhao from Shantou University. *V. parahaemolyticus* ATCC17802 was purchased from Guangdong Huankai Microbial Technology Co., Ltd. *E. tarda* was incubated in tryptic soy broth (TSB) at 37 °C for 16 h [66] (HuanKai Microbiology Technology Co., Ltd., Guangdong, China) with oscillation at 220 rpm. *A. hydrophila* Ah-HN1 was cultured in Luria Broth (LB) medium for 16 h, whereas *V. alginolyticus* ATCC33787 and *V. parahaemolyticus* ATCC17802 were cultured in LB medium for 12 h.

### Ethics statement

All animal experiment protocols were reviewed and approved by the Laboratory Animal Welfare and Ethics Committee of Jinan University (Approval No. IACUC-20240507-14). All procedures, including euthanasia, were conducted in compliance with the AVMA Guidelines for the Euthanasia of Animals (2020) and the National Institutes of Health Guide for the Care and Use of Laboratory Animals. All animal procedures followed the ARRIVE guidelines 2.0 (the complete checklist is available at Figshare: https://doi.org/10.6084/m9.figshare.30085792).

### Animal culture

Nile tilapia (*Oreochromis niloticus*), were purchased from Guangzhou Tilapia Farming Base (Guangzhou, China), hatched from the same batch of fertilized eggs, and collected at one month post fertilization (weight of 30 ± 0.2 g), with equal numbers of males and females. The fish were reared in 80-L tanks equipped with closed recirculating aquaculture systems and fed a commercial fish diet. Physicochemical parameters were maintained as follows: water temperature, 28 °C; dissolved oxygen, 6–7 mg/L; carbon dioxide content, 10 mg/L; pH value, 7.0–7.5. The animals were acclimated to the experimental conditions for two weeks prior to the experiment. Fish were fed twice daily with commercial fish feed under a 12-h light/dark cycle. The tank was cleaned twice daily using a siphon to remove food debris and feces, ensuring a clean and healthy environment.

### Antimicrobial assay

Single colonies were picked and inoculated in 30 mL of TSB medium and shaken at 220 rpm overnight at 37 °C until saturated. After centrifugation at 8, 000 g for 5 min to remove the supernatant, the cells were washed three times with sterile saline and resuspended in M9 medium (containing 10 mM CH_3_COONa, 1 mM MgSO_4_, and 100 μM CaCl_2_, from Sangon Biotech). Next, the OD_600_ value was adjusted to 0.2, and indole (Aladdin, I811715-25 g), florfenicol (Aladdin, 73,231–34-2), and/or other chemicals (carbonyl cyanide m-chlorophenylhydrazone (Sigma), FeSO_4_, ammonium ferric citrate, succinic acid from Sangon Biotech) were simultaneously added to the M9 medium and the culture was incubated at 37 °C and 220 rpm for 6 h. Finally, 100 μL of bacterial culture was aspirated for serial dilution. Aliquots (10 μL) of each culture were inoculated on TSB agar. After 16 h, when bacteria had grown into individual colonies, the samples were used to determine bacterial counts.

### Minimum inhibitory concentration determination

The minimum inhibitory concentration (MIC) was tested according to the Clinical Laboratory Standards Institute [67]. Individual colonies were picked and cultured in TSB broth medium until saturated. Saturated bacteria were inoculated into fresh liquid medium at a ratio of 1:100 and incubated until the OD_600_ was 0.5–0.8. The bacterial solution was then diluted 100-fold using Mueller – Hinton broth (MHB) (Huankai, China) medium to obtain a colony count of approximately 5 × 10^6^. 160 μL of broth was added to the first well of the 96-well plate, and 90 μL was added to each of the remaining wells. Then, 20 μL of antibiotic was added to the first well and mixed by pipetting, with 90 μL was pipetted into the next well and mixed. This was repeated until the last well, after which diluted bacterial solution (10 μL) was added to each well. Finally, the 96-well plate was incubated in an incubator at 37 °C for 16 h to observe bacterial growth. The antibiotic concentration corresponding to the wells with invisible colonies was considered as the MIC of the antibiotic.

### Metabolomics and data analysis

Five single colonies were inoculated into 30 mL of TSB and incubated at 37 °C and 220 rpm for 16 h until saturation. Cells were collected by centrifugation, washed three times with 0.85% NaCl to completely remove TSB, and then resuspended in M9 medium to OD_600_ = 1.0; after adding 2 mM indole, the mixture was incubated for 6 h at 37 °C. After 6 h of incubation, bacteria were collected by centrifugation at 4 °C and 12, 000 g then washed three times with PBS. The collected cells were quenched with liquid nitrogen, mixed with 1 mL of extract containing deuterated internal standard (MeOH:ACN:H_2_O, 2:2:1 (v/v/v)) for 30 s, homogenized for 4 min at 35 Hz, and then transferred to an ice water bath to sonicate the samples for 5 min; this process was repeated three times. The samples were then thawed at room temperature and mixed for 30 s. This process was repeated three times. The samples were then sonicated in a water bath at 4 °C for 10 min then incubated at − 40 °C for 1 h to precipitate proteins. The samples were centrifuged at 12, 000 g at 4 °C for 15 min and the resulting supernatant was transferred to a fresh glass vial for analysis.

For polar metabolites, liquid chromatography (LC) analysis was performed using an ultra-high performance liquid chromatography (UPLC) system (Vanquish, Thermo Fisher Scientific) and Waters ACQUITY UPLC BEH Amide (2.1 mm × 50 mm, 1.7 μm) coupled to an Orbitrap Exploris 120 mass spectrometer (Orbitrap MS, Thermo). The mobile phase comprised an aqueous solution of 25 mmol/L ammonium acetate and 25 ammonia hydroxide (pH = 9.75) (phase A) and acetonitrile (phase B). The autosampler temperature was 4 °C and the injection volume was 2 μL. The Orbitrap Exploris 120 mass spectrometer was used for its ability to acquire MS/MS spectra in Information Dependent Acquisition (IDA) mode under the control of acquisition software (Xcalibur, Thermo). In this mode, the acquisition software continuously evaluates the full-scan MS spectra. The electrospray ionization source conditions were set as follows: sheath gas flow rate, 50 Arb; auxiliary gas flow rate, 15 Arb; capillary temperature, 320 °C; full MS resolution, 60, 000; MS/MS resolution, 15, 000; collision energies, SNCE 20/30/40; and spray voltages, 3.8 kV (positive) or −3.4 kV (negative). The raw data were converted to mzXML format using ProteoWizard and processed using an in-house programme, developed using R and is based on XCMS for peak detection, extraction, alignment, and integration. The R package and BiotreeDB (V3.0) were applied for metabolite identification [68].

Metabolomics data analysis was performed using IBM SPSS Statistics (version 22.0; SPSS Inc., Chicago, IL, USA). Values with *p* < 0.05 were considered statistically significant. Hierarchical cluster analysis was performed using R Studio software (version 4.0.3). SIMCA – P 14+ (Umetrics, Sweden) was used to analyze the principal components and orthogonal partial least squares. Z-scores were analyzed in Microsoft Excel by highlighting significant deviations from the mean. Metabolic pathway enrichment and metabolic network analysis were performed using MetaboAnalyst (version 6.0; http://www.metaboanalyst.ca/) and iPath (version 3.0; https://pathways.embl.de/). Graphs were plotted using Microsoft Excel and GraphPad Prism 8.0 (San Diego, CA, USA).

### Quantitative reverse-transcription PCR analysis

Quantitative reverse-transcription PCR (RT-qPCR) was performed as previously described, with some modifications [69]. Specifically,bacterial cells (1.5 mL) were harvested at OD_600_ = 1.0, centrifuged (12, 000 g, 4 °C, 3 min) and immediately quenched in liquid nitrogen. Cells were then lysed for total RNA extraction using TRIZOL reagent (Invitrogen Life Technologies, USA) according to the manufacturer’s protocols, and total RNA was extracted using the Evo M-mLV RT Mix Kit with gDNA Clean for qPCR (Accurate Biotechnology, Hunan, China). According to the manufacturer’s instructions. Ltd., Guangdong, China, 1 μg of total RNA was used for RT-qPCR, which was performed in 96-well plates, with each well containing a total volume of 10 μL of liquid, comprising 5 μL of 2× SYBR Green Pro Taq HS premix (Accurate Biology, China), 4 μL of cDNA template, and 0.5 μL of each primer (10 μM). Primer sequences are listed in Table S1-S3. Three biological replicates were used, according to the manufacturer’s instructions, and all assays were performed using the CFX Connect Real-Time System (CFX96; Bio-Rad, USA). Cycling parameters were as follows: initial denaturation of the target gene at 95 °C for 30 s, 45 cycles at 95 °C for 5 s, and 30 s at 58 °C. mRNA levels were normalized to the 16S rRNA gene, which was constitutively and stably expressed under the analytical conditions.

### Enzyme activity assay

Enzyme activity was determined as previously described [70]. Cultured bacterial cells were harvested and resuspended in M9 medium to an OD_600_ of 1.0. Samples (10 mL) were collected by centrifugation at 8, 000 g for 5 min. Cells were then resuspended in PBS and lysed by sonication on ice (200 W total power, 50% output, 2-s pulse and 3- s pause) for 3 min, followed by centrifugation at 12, 000 g for 10 min. The resulting supernatant was used for the enzyme assay. Enzyme activity was determined spectrophotometrically by monitoring the reduction of 3-(4,5-dimethyl-2-thiazolyl)-2,5-diphenyl-2 H-tetrazolium bromide (MTT) (CAS: 298–93-1, Sigma) at 566 nm. Protein concentration was determined using the BCA assay (Beyotime, Shanghai, China) after centrifuging the samples at 12, 000 g for 10 min.

The reaction mixtures were as follows. The α-Ketoglutarate dehydrogenase (OGDH) reaction mixture contained 0.5 mM MTT, 50 mM potassium phosphate buffer, 2.5 mM MgCl_2_, 0.5 mM phenazine methyl sulfate (PMS) (CAS: 299–11-6, Sigma), 0.2 mM thiamine pyrophosphate (CAS: 154–87-0, Aladdin), and 10 mM potassium α-ketoglutarate; The isocitrate dehydrogenase (ICDH) reaction mixture contained 0.5 mM MTT, 0.5 mM PMS, 2.5 mM MgCl_2_, 100 mM Tris-HCl [pH 8.8], and 7 mM isocitrate. The malate dehydrogenase (MDH) reaction mixture contained 0.5 mM MTT, 1 mM PMS, 2.5 mM MgCl_2_, 50 mM potassium phosphate buffer, and 80 mM sodium malate. The pyruvate dehydrogenase (PDH) reaction mixture contained 0.5 mM MTT, 0.5 mM PMS, 2.5 mM MgCl_2_, 0.2 mM thiamine pyrophosphate, 50 mM potassium phosphate buffer, and 0.16 M sodium pyruvate. The supernatant containing 200 μg of total protein was added to the reaction mixture and the final volume of 200 μL was made up in a 96-well plate. The plates were then incubated at 37 °C for 20 min in the dark and the absorbance was measured by colorimetric reading at 566 nm. Citrate synthase activity was measured using the Citrate Synthase Assay Kit (Cominbio, Suzhou, China). 3-phosphoglyceraldehyde dehydrogenase activity was determined using the GAPDH enzyme activity kit (Qiyi Biotechnology Co., LTD., China).

### NADH content measurement

A NAD^+^/NADH Assay Kit (S0175, Beyotime, China) was used to measure NADH content. Cultured bacterial cells were harvested and resuspended in M9 medium to an OD_600_ of 0.2, then incubated 2 mM indole with or without 100 μg/mL florfenicol for 6 h. Samples (10 mL) were collected by centrifugation at 8, 000 g for 5 min. Cells were then resuspended in PBS and lysed by sonication on ice (200 W total power, 50% output, 2-s pulse and 3-s pause) for 3 min, followed by centrifugation at 12, 000 g for 10 min. The resulting supernatant was used to determine the NADH content determination.

### ATP levels measurement

ATP levels were determined using the BacTiter-Glo™ microbial cell viability assay (Promega, USA). After 16 h cultures were harvested and resuspended to an OD600 of 0.2 in M9 medium, and incubated 2 mM indole with or without 100 μg/mL florfenicol for 6 h. Added 50 μL aliquot of the bacterial suspension and an equal volume of test solution were mixed in a black 96-well plate and gently shaken. After a 5 min dark reaction, fluorescence was detected using an enzyme-labeled instrument (Biotek, Synergy HT, Vermont, USA).

### ROS assay

ROS levels were detected using 2“, 7”-dichlorodihydrofluorescein diacetate (DCFH-DA) (Sigma). Overnight bacterial cultures were collected by centrifugation at 8, 000 g for 5 min. After three washes with 0.85% sterile saline, the bacteria were resuspended in M9 basal medium to an OD_600_ of 0.2. Next, 2 mM indole or/and 100 μg/mL florfenicol were added to the medium and the mixture was incubated at 37 °C and 220 rpm for 6 h. Then, 196 μL of culture and 4 μL of DCFH-DA were added to a 96-well plate and incubated at 37 °C for 30 min in the dark. Fluorescence units were immediately measured at excitation and emission wavelengths of 485 nm and 515 nm, respectively, using a fluorescence enzyme marker (Biotek, Synergy HT, Vermont, USA).

### Ferrous ion content measurement

Ferri ion in the samples were detected using the Ferrous Ion Assay Kit (Cat: BC5415, Solebo, Beijing, China). Cultured bacterial cells were harvested and resuspended in M9 medium to an OD_600_ of 1.0. Samples (10 mL) were collected by centrifugation at 8, 000 g for 5 min. Cells were resuspended in PBS and lysed by sonication on ice (200 W total power, 50% output, 2-s pulse and 3-s pause) for 3 min, followed by centrifugation at 12, 000 g for 10 min. The resulting supernatant was used to determine of ferrous ion content.

### Efflux pump activity measurement

The efflux pump activity was determined using ethidium bromide (Macklin, Shanghai, China). Overnight bacterial cultures were collected by centrifugation at 8, 000 g for 5 min. After washing three times with 0.85% sterile saline, the bacteria were resuspended in M9 basal medium to an OD_600_ of 0.2, after which 2 mM indole and/or 100 μg/mL florfenicol were added to the medium, which was incubated for 6 h at 37 °C and 220 rpm. Then, 190 μL of culture and 10 μL of 20 mM ethidium bromide were added to a 96-well plate and incubated for 1 h at 37 °C in the dark. After incubation, fluorescence units were measured using a fluorescence enzyme marker (Biotek, Synergy HT, Vermont, USA) at excitation and emission wavelengths of 530 nm and 600 nm, respectively.

### Intracellular florfenicol content measurement

A florfenicol ELISA Kit (JM-06696O1, Jingmei, Jiangsu, China) was used to determine the intracellular florfenicol content. Cultured bacterial cells were harvested and resuspended in M9 medium to an OD_600_ of 1.0 and treated with 2 mM indole and/or 100 μg/mL florfenicol. Samples (10 mL) were collected by centrifugation at 8, 000 g for 5 min. Cells were resuspended in PBS and lysed by sonication on ice (200 W total power, 50% output, 2-s pulse and 3-s pause) for 3 min, followed by centrifugation at 12, 000 g for 10 min. The resulting supernatant was used to determine the florfenicol content.

### Intracellular pH measurement

Intracellular pH was measured using carboxyfluorescein succinimidyl ester (CFSE) (YuanYe, China). Bacteria were resuspended in M9 medium to OD_600_ = 0.2. Then, 2 mM indole and/or 100 μg/mL florfenicol were then added to the medium and the mixture was incubated at 37 °C and 220 rpm for 6 h. After 6 h, 198 μL of culture was aspirated, 2 μL of CFSE was added and mixed in a 96-well plate to give a final CFSE concentration of 100 μM, and the culture was incubated for 1 h at 37 °C in the dark. Fluorescence was then measured at excitation and emission wavelengths of 500 nm and 520 nm, respectively, using a fluorescence enzyme marker (Biotek, Synergy HT, Vermont, USA).

### Membrane potential measurements

Membrane potential was measured using DiBAC_4_(3) (Glpbio, China) as previously described [71]. Briefly, we collected the bacteria from overnight cultures, adjusted their OD_600_ value to 0.2 and incubated the bacteria in M9 medium with/without 2 mM indole and 100 μg/mL florfenicol for 6 h at 37 °C and 220 rpm. Then, 198 μL of culture was mixed with the DiBAC_4_(3) and brought to a final concentration of 100 μM in a total volume of 200 μL, then incubated for 1 h at 37 °C in the dark. Finally, fluorescence was measured at excitation and emission wavelengths of 490 nm and 516 nm, respectively, using a fluorescence enzyme marker (Biotek, Synergy HT, Vermont, USA).

### Membrane permeability measurement

Membrane permeability was determined using 1-N-Phenylnaphthyridine (CAS: 90–30-2, Aladdin) solution. Samples were centrifuged at 8, 000 g centrifugation for 5 min was used to collect overnight bacterial cultures. After washing three times with 0.85% sterile saline, the bacteria were resuspended in M9 basal medium to an OD_600_ of 0.2. After which 2 mM indole and/or 100 μg/mL florfenicol were added to the medium, which was incubated for 6 h at 37 °C and 220 rpm. Then, 198 μL of culture and 2 μL of 20 mM 1-N-Phenylnaphthyridine solution were mixed and added to a 96-well plate and incubated for 1 h at 37 °C in the dark. Fluorescence units were then measured using a fluorescence enzyme marker (Biotek, Synergy HT, Vermont, USA) at excitation and emission wavelengths of 340 nm and 420 nm, respectively.

### Transmission electron microscope analysis

*E. tarda* ATCC15947 was cultured overnight in 30 mL of TSB broth at 37 °C and 220 rpm. The next day, bacteria were collected by centrifugation and resuspended in M9 medium to OD_600_ = 0.2; after adding 2 mM indole and 100 μg/mL florfenicol, the mixture was incubated at 37 °C for 6 h. After incubation, bacteria were collected by centrifugation, washed three times with PBS, then fixed by electron microscopy for 2 h at room temperature. The fixed samples were drifted in 0.1 M phosphate buffer (pH = 7.4) three times for 15 min each time. The samples were then transferred to 1% OsO_4_ (CAS: 20,816–12-0, Sigma) in 0.1 M phosphate buffer for 1–2 h at room temperature to complete post-fixation then dehydrated with increasing concentrations of ethanol (30%, 50%, 70%, 80%, 90%, 95%, 100%) and isoamyl acetate (v : v = 1 : 1) for 15 min [72]. Finally, the samples were freeze-dried and gold-plated. The ultrastructure was observed using a transmission electron microscopy (Hitachi SU8100; Hitachi, Japan). The experiment was conducted with three independent biological replicates.

### Nile tilapia infection and hematoxylin-eosin staining

The *E. tarda* ATCC15947 monoclonal strain was cultured overnight at 37 °C and 220 rpm with shaking. The next day, the sample was centrifuged at 8, 000 g for 5 min to collect the bacteria, which were washed three times with saline, and then prepared into a bacterial suspension with saline for injection into the fish at a dose of 100 μL bacterial suspension (1 × 10^9^ CFU/mL). A total of 120 fish were injected and randomly divided into four groups, each consisting of 30 fish. Group 1 was treated with saline (control), group 2 was treated with indole (10 mg/kg), group 3 was treated with florfenicol (15 mg/kg), and group 4 was treated with florfenicol plus indole (15 mg/kg florfenicol +10 mg/kg indole). To simulate the scenario of discovering severe infections followed by treatment in some practical environments, we designed an experiment where fish were subjected to a high-dose bacterial infection. One hour after infection, fish were administered indole and/or florfenicol. After 24 h of treatment, the whole liver and spleen of the Nile tilapia were collected, weighed, homogenized, and counted on a spot plate. Simultaneously, the liver and spleen were removed for hematoxylin-eosin staining, fixed with 10% formalin in PBS, and then sectioned after ethanol dehydration and paraffin embedding. The sections were dewaxed with xylene and alcohol in turn, and the xylene was removed, stained with hematoxylin and eosin, dehydrated with alcohol, and finally sealed with neutral gum and photographed. The number of surviving fish in each group was enumerated daily. By the 7th day of observation, the survival rate had stabilized. The Nile tilapia in the control and experimental groups were observed continuously for seven days. Finally, the fish were euthanized via intramuscular injection of MS-222 finally.

### Motility assay

Overnight bacterial cultures were collected by centrifugation at 8, 000 g for 5 min. After washing three times with 0.85% sterile saline, the bacteria were resuspended in M9 basal medium to an OD_600_ of 0.2, after which 2 mM indole or/and 100 μg/mL florfenicol were added to the medium, which was incubated for 6 h at 37 °C and 220 rpm. Next, 10 μL of the bacterial solution was placed in the center of TSB agar plates (0.3% and 0.6%), which were incubated in the incubator at 37 °C for 16 h. Then, we observed the size and morphology of the bacterial circle in the two plates, which reflect the swimming and swarming motility of the bacteria (0.3% and 0.6% TSB, respectively).

### Oxford cup assay

Single colonies were picked and incubated it in TSB broth medium overnight until saturated. The next day, the saturated bacteria was inoculated into fresh liquid medium at a ratio of 1:100 and incubated for 2 h to an OD_600_ of 0.5–0.8. Then, 500 μL of bacterial solution was spread uniformly on the TSB agar plate, which was allowed to dry. A sterile oxford cup (dimensions: 8 mm × 6 mm × 10 mm, Biosharp, China) was placed on the agar plate, to which we added 50 μL of florfenicol and/or indole to a final concentration of 12.5/25/50/100/200 μg/mL (florfenicol) and 2 mM (indole). This was followed by incubation at 37 °C for 16 h, after which the size of the ring of inhibition was observed.

## Statistical analysis

Statistical analysis and data visualization were performed using GraphPad Prism 8.0 for data analysis and visualization. Following an assessment of data normality, pairwise comparisons were conducted using a paired two-tailed Student’s t-test, unless otherwise specified. Results were presented as means ± standard error of the mean (SEM). Each experiment was performed with at least three independent biological replicates to ensure robust and reliable outcomes. Statistical significance is denoted in the figure legends as follows: * indicates *p* < 0.05, ** denotes *p* < 0.01, *** denotes *p* < 0.001, and ns indicates no significant difference.

## Supplementary Material

Figure S2.jpg

Clean Copy of Supplementary Material - QVIR-2025-0761.R1.docx

Figure S1.jpg

Figure S3.jpg

Figure S4.jpg

Tables.docx

## Data Availability

The metabolomic data & Author checklist were deposited to Figshare (https://doi.org/10.6084/m9.figshare.30085792) [73].
