# Supplementary figures and images for: Exogenous indole promotes florfenicol tolerance in Edwardsiella tarda
Source: Virulence. 2026 Jan 21;17(1):2620188. doi: 10.1080/21505594.2026.2620188 (PMC12834173; doi:10.1080/21505594.2026.2620188)

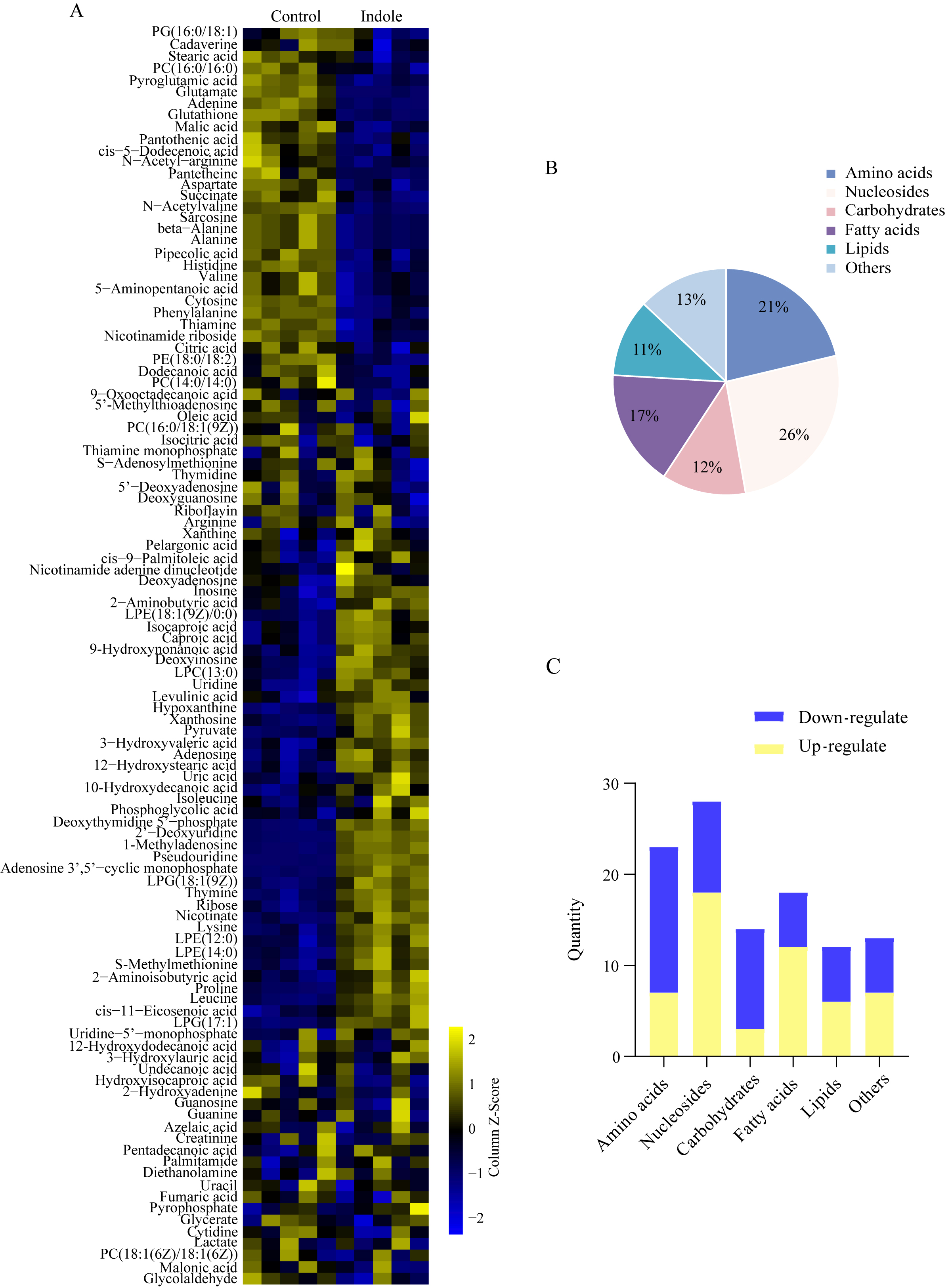

Supplement: Figure S2.jpg [file KVIR_A_2620188_SM6319.jpg]

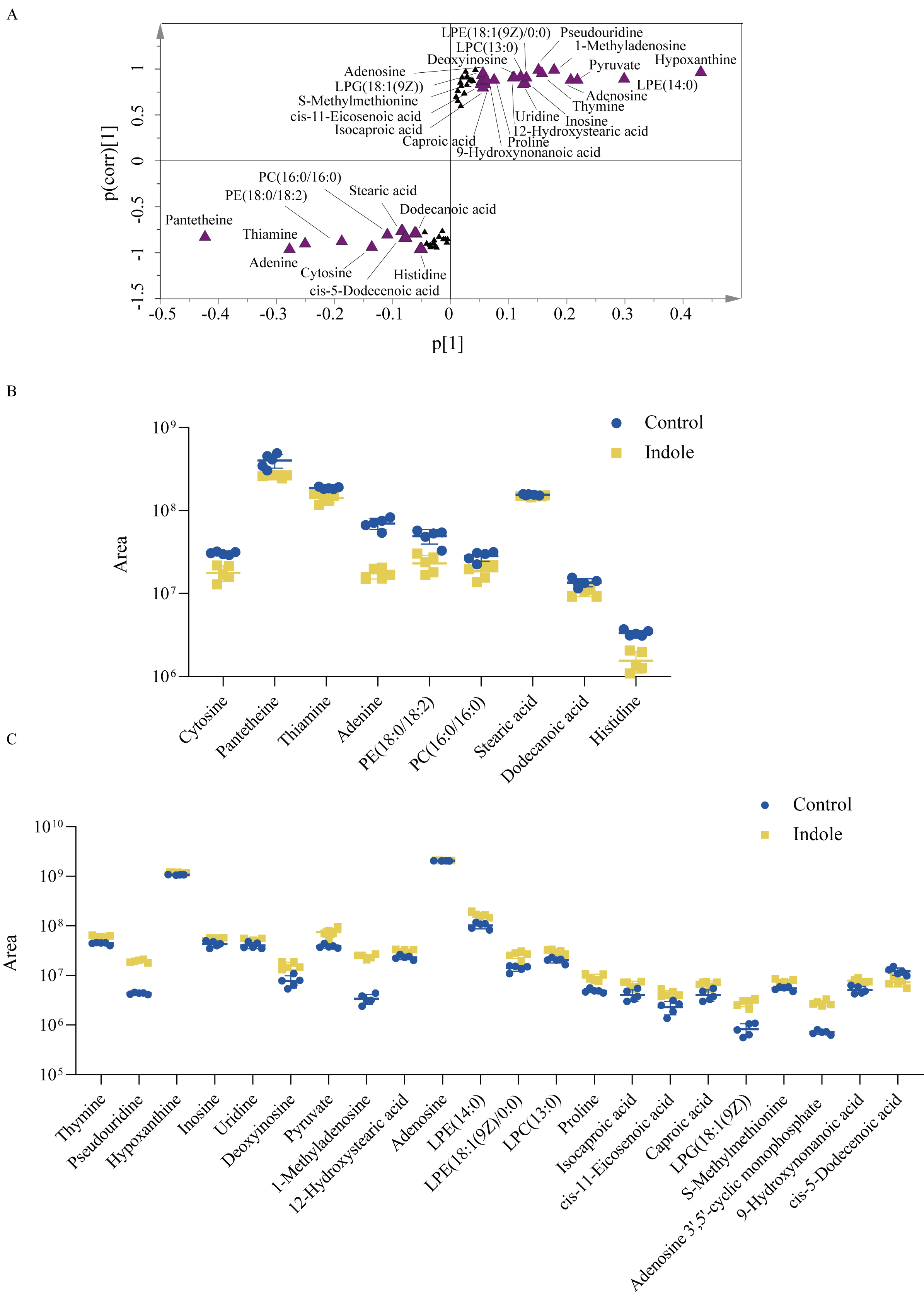

Supplement: Figure S3.jpg [file KVIR_A_2620188_SM6316.jpg]

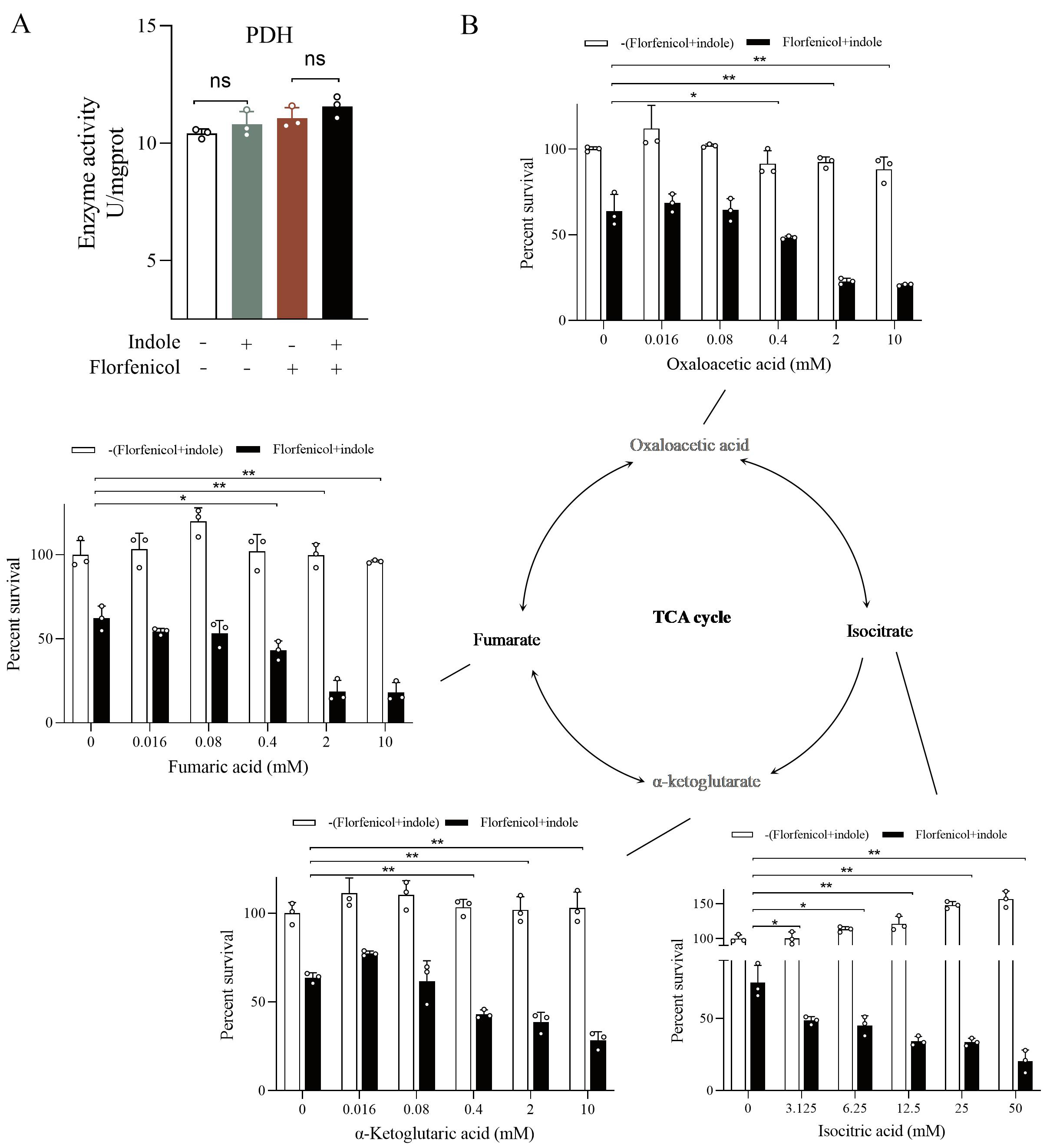

Supplement: Figure S4.jpg [file KVIR_A_2620188_SM6315.jpg]
