# Supplementary material for: Exogenous indole promotes florfenicol tolerance in Edwardsiella tarda
Source: Virulence. 2026 Jan 21;17(1):2620188. doi: 10.1080/21505594.2026.2620188 (PMC12834173; doi:10.1080/21505594.2026.2620188)
Supplement: Tables.docx [file KVIR_A_2620188_SM6314.docx]

Table S1 qRT-PCR primers for TCA cycle-related genes

| Gene | 5’- 3’ | Sequences |
| --- | --- | --- |
| 16S rRNA | Forward | actgagacacggtccagactcctac |
|  | Reverse | ttaacacgcacaccttcctccctac |
| *gltA* | Forward | acgcaggcagagtatgacac |
|  | Reverse | tgtgggtagataaagggttgcc |
| *acnB* | Forward | ctgaaggttgaacaggctttcg |
|  | Reverse | aataggctccttgatgctgtcg |
| *icd* | Forward | tggcgatggcatactcgatc |
|  | Reverse | tacgccggtatcgaatggaag |
| *sucA* | Forward | gttaaagctctggccgaaacg |
|  | Reverse | atgaaggcctggctggattc |
| *sucB* | Forward | tatcgacatcgcggatcacag |
|  | Reverse | gtgtccgtttgggctttatgtc |
| *sucC* | Forward | ggaacagagcattactgtcgac |
|  | Reverse | tggctttcaaactgggtctg |
| *sucD* | Forward | ttcaccagcagcatgtcctg |
|  | Reverse | gcgccagcgtgatttatgtc |
| *frdA* | Forward | acgttcaaaccatgctccag |
|  | Reverse | accaggaaggcaacgaaaac |
| *frdB* | Forward | tttcgggcaaacttctgagc |
|  | Reverse | tgcatcaactgtggtctgtg |
| *frdC* | Forward | ttgcaggaaagcgacaaagc |
|  | Reverse | tgacgacttcaaccaaacgc |
| *frdD* | Forward | atgctctggctgaagctcag |
|  | Reverse | atgtggggtgccatctttgc |
| *sdhA* | Forward | tattgacgtggcgttgtacc |
|  | Reverse | cgcaaggtttggctgaattg |
| *sdhB* | Forward | tttcgggcaaacactgacac |
|  | Reverse | gttggatgggctgtatgaatgc |
| *sdhC* | Forward | gcggataccaccacaaatatgg |
|  | Reverse | ttccccattactgccattgc |
| *sdhD* | Forward | agggtaaacacctgggtcaac |
|  | Reverse | tggttactgttgcgtgcatc |
| *fumB* | Forward | tcagtttttccggcgtcaac |
|  | Reverse | taacctgcgttactcccagaac |
| *fumC* | Forward | gcggtatgatgcggaaagttc |
|  | Reverse | aatgggttcggcctatcttca |
| *mdh* | Forward | acgcctgtatcggtatcatcac |
|  | Reverse | agatcggtcacttcttgctcag |

Table S2 qRT-PCR primers for NADH dehydrogenase genes

| Gene | 5’- 3’ | Sequences |
| --- | --- | --- |
| *nuoA* | Forward | tcgccgtttttgttctcgtc |
|  | Reverse | aaccagccgttttcacgtac |
| *nuoB* | Forward | cttgcaccaccgaatagatgtc |
|  | Reverse | tcaactgggggcgtaaaaac |
| *nuoC* | Forward | atccaactgcaacacgatgc |
|  | Reverse | acaccccctgcgtaaagattac |
| *nuoD* | Forward | ttcgcatcaacagccatctg |
|  | Reverse | tggaacgccctttcagaatg |
| *nuoE* | Forward | accacactgtcacaatagcg |
|  | Reverse | aggattcggttttcgtgctc |
| *nuoF* | Forward | gctgctcaagggtttcgatatc |
|  | Reverse | atttcgccagcatcggtaag |
| *nuoG* | Forward | tcacggtgccattaaaacgg |
|  | Reverse | aagtgggacatgcagtttgc |
| *nuoH* | Forward | cgcgtaccagaatgaacatcac |
|  | Reverse | gcttcttgaccttcatcatcgc |
| *nuoI* | Forward | acacatgccacagaagatgc |
|  | Reverse | tcggctgtatttcgttgcag |
| *nuoJ* | Forward | tgatgttcacgaccgatgtg |
|  | Reverse | tcgtggtgatgatgctcaac |
| *nuoK* | Forward | cgccaagatatacatcacctgac |
|  | Reverse | cggcgtaacctgctgtttatg |
| *nuoL* | Forward | acagcaacgccttgaagaag |
|  | Reverse | accatgagccagatcggttac |
| *nuoM* | Forward | tgaaacgcagcagaccatag |
|  | Reverse | atgttgatcgccatcttcgg |
| *nuoN* | Forward | tgaacagacgcaacaacacc |
|  | Reverse | atcgtttgacacagccgttg |

Table S3 Primers used in this study

| Gene | 5’- 3’ | Sequences |
| --- | --- | --- |
| *oxyR-1* | Forward | ccgtgttgcgcgaggtcaag |
|  | Reverse | gaaggcctggtgtagcatggg |
| *oxyR-2* | Forward | ggatgcgtcggcggcgaaaccattg |
|  | Reverse | cggcgcggcagatctggctattgcc |
| *rpoS* | Forward | cccctccacttggatttgcctcacc |
|  | Reverse | gctgcgtcttaacgagcgtatcacc |
| *sodB* | Forward | cgatcaacgcggcctttgg |
|  | Reverse | cagcagcggcttgtcttcac |
| *sodC1* | Forward | ggcctatgccgcctcactca |
|  | Reverse | ggcagctggcgttctcatg |
| *katG* | Forward | ggcacgcaggatagagttgg |
|  | Reverse | gaagccgaccggtagcaatc |
| *fur* | Forward | gtcacgcttccccgtctcaag |
|  | Reverse | gactgacttaccgccctcgaag |
| *feoB* | Forward | ccagttgaccggcgcacgtc |
|  | Reverse | gctgcgggcgatgtccaac |
| *feoC* | Forward | ccgttacgttgcatggacgc |
|  | Reverse | gcttcggggcagcctttg |
| *emrA* | Forward | cccccgccccgaataagaag |
|  | Reverse | aatctgcaccaggttgccgg |
| *emrB* | Forward | cccgaccatctcgggtaact |
|  | Reverse | tccaggctctgcgagatccc |
| *mdfA* | Forward | cagattgaaggcaccgttac |
|  | Reverse | atgagtaaggggacagtctc |
| *ycaD* | Forward | ccagtgatcggaaaagcgctgcatc |
|  | Reverse | cggcagcctggctatgttggttagc |
| *bmr3* | Forward | cgatcagcgcggccgcgatgaatac |
|  | Reverse | gcgatcctcggggcaaccctcaacc |
| *ompA* | Forward | ctgagctacccgctgatgg |
|  | Reverse | ctgctggtgccgatcttgttg |
| *ompW* | Forward | cgaactggtcgcctggagat |
|  | Reverse | acgccattccgccacaag |
